# Pigment production by a newly isolated strain *Pycnoporus sanguineus* SYBC-L7 in solid-state fermentation

**DOI:** 10.3389/fmicb.2022.1015913

**Published:** 2022-10-19

**Authors:** Di Meng, Xuan Shao, Shou-Peng Luo, Qiao-Peng Tian, Xiang-Ru Liao

**Affiliations:** ^1^Henan Provincial Engineering Research Center for Development and Application of Characteristic Microorganism Resources, Shangqiu Normal University, Shangqiu, China; ^2^Hua Tian Engineering & Technology Corporation, MCC, Nanjing, China; ^3^Key Laboratory of Industrial Biotechnology, Ministry of Education, School of Biotechnology, Jiangnan University, Wuxi, China

**Keywords:** natural pigment, food- and cosmetic-grade microorganism, *Pycnoporus sanguineus*, agro-industrial wastes, cinnabarinic acid

## Abstract

Natural pigments are playing important roles in our daily lives. They not only make products colorful but also provide various health benefits for humans. In addition, *Pycnoporus* genus, listed as food- and cosmetic-grade microorganism, is one of the promising organisms for developing natural pigments. In this study, a new fungal strain with high efficiency in producing intense orange pigments was isolated and identified as *Pycnoporus sanguineus* SYBC-L7. Different agro-industrial wastes were applied to evaluate the growth and pigment production of strain SYBC-L7. SYBC-L7 can grow rapidly and effectively produce pigments using wood chips as substrate in solid-state fermentation (SSF). Culture conditions were also optimized for value-added pigments production and the optimum production conditions were glucose as carbon source, ammonium tartrate as nitrogen source, initial pH 6.0, and relative humidity of 65%. Pigment components, cinnabarinic acid, tramesanguin, and 2-amino-9-formylphenoxazone-1-carbonic acid were confirmed by liquid chromatography–mass spectrometry. Meanwhile, an agar plate diffusion assay was performed to evaluate the antimicrobial activity of the pigment. These pigments showed more significant inhibition of Gram-positive than Gram-negative bacteria. The results showed that *Pycnoporus sanguineus* SYBC-L7 was able to cost-effectively produce intense natural orange pigments with antibacterial activity in SSF, which is the basis of their large-scale production and application.

## Introduction

Pigments have become an important part of cosmetics, food, textiles, and other industrial fields (Meruvu and dos Santos, 2021). Some of them not only endow products with different colors but also have antibacterial and antioxidant activity to provide various health benefits for humans (Tudor et al., 2013; Srivastava et al., 2022). According to the source, pigments can be divided into synthetic pigments and natural pigments. Since synthetic pigments are found to display potential toxicity, carcinogenicity, and undesirable side effects on human health and the environment, natural pigments (derived from plants, animals, or microorganisms) are getting more attention due to their biodegradability, no side effects, and biological activities (Chatragadda et al., 2019; Darwesh et al., 2020; Chatragadda and Dufosse, 2021). Among natural pigment producers, microorganisms are noteworthy for their all-seasonal production of stable and low-cost with high yield (Meruvu and dos Santos, 2021). To date, many microorganisms have been reported to produce pigments, including *Monascus* sp. (Chen et al., 2021), *Penicillium* sp. (Meruvu and dos Santos, 2021), *Pycnoporus* sp. (Tellez-Tellez et al., 2016; Zhang et al., 2019), *Rhodotorula* sp., and *Bacillus* sp. (Usmani et al., 2020). Among them, *Pycnoporus* genus is a white-rot fungus, listed as food- and cosmetic-grade microorganism, and one of the promising organisms for the development of natural pigments (Lomascolo et al., 2011; Tellez-Tellez et al., 2016). It has been reported that the major *Pycnoporus* pigments (cinnabarin, cinnabarinic acid, and tramesanguin), possessing antiviral, antibacterial, and anti-inflammatory properties, are derived from a phenoxazine-3-one type structure, which is the central core of many natural active compounds (Tellez-Tellez et al., 2016; Zhang et al., 2019). Furthermore, *Pycnoporus* strains also can produce various useful enzymes for industry, including hydrolases and oxidases mainly laccases, which make them easier to use agro-industrial wastes (Tellez-Tellez et al., 2016).

Solid-state fermentation (SSF) is a traditional fermentation method of fungi to produce pigments. SSF provides attachment sites for the growth of strains, sufficient nutrients, and oxygen supply, making the yield of pigments produced much higher than that of liquid submerged fermentation (Palma et al., 2016; Feng et al., 2022). At present, various agro-industrial wastes (sugarcane bagasse, sawdust, rice straw, wheat straw, orange peel, and so on) are used as substrates for SSF, which can help to reduce the production cost, reduce the pollution load from the environment, and create maximum value (Sadh et al., 2018). In addition, some fermentation conditions like pH, temperature, relative humidity, and media nutrients also affect the production of pigment. Therefore, exploiting agro-industrial wastes as substrates and optimizing fermentation conditions will be important for value-added metabolite production.

Although *Pycnoporus* genus has been used to produce high value-added metabolites through solid-state fermentation, few studies on agro-industrial wastes as substrates for pigment production have been reported at present. Therefore, the aims of this paper were to (1) isolate and identify a *Pycnoporus* fungus with high-yield pigment production capacity; (2) improve pigment production by controlling culture conditions, including substrates, carbon and nitrogen sources, initial pH, and relative humidity; (3) analyze and identify the pigments; and (4) evaluate the antimicrobial activity of pigments.

## Materials and methods

### Materials

Cinnabarinic acid (CA, ≥98%, CAS: 606–59-7), Ehrlich reagent (CAS: 100–10-7), and N-acetylglucosamine (CAS: 7512-17-6) were purchased from Sigma-Aldrich (Shanghai, China). Sugarcane bagasse, wheat straw, rice straw, water hyacinth, and wood chip were purchased from a local market and dried to constant weight at 60°C before use. Other chemical reagents, unless otherwise specified, were all analytical grade.

Indicator strains, including *Bacillus subtilis* SYBC-hb1, *Bacillus subtilis* SYBC-hb5, *Bacillus licheniformis* SYBC-hb2, *Bacillus pumilus* SYBC-hb4, *Bacillus thuringiensis* SYBC-hb7, *Bacillus amyloliquefaciens* Y1-A3, *Bacillus megaterium* H021-A1, *Bacillus cereus* SYBC-hb8, *Lysinibacillus* sp. H021-S8, *Staphylococcus kloosii* H008-B4, *Staphylococcus aureus*, *Escherichia coli* J159, *Serratia* sp. L1, *Serratia* sp. L2, and *Serratia* sp. L3, were obtained from Biocatalysis and Transformation Biology lab at Jiangnan University (Wuxi, China).

### Isolation samples

Ten fungal fruit body samples were collected from different locations in a local forest in Wuxi, China (31°32′24′′N, 120°12′24′′E).

### Cultivation medium

Potato dextrose agar (PDA) was prepared for purification and preservation.

Seed medium was prepared as described by Zeng et al. (2011) with certain modifications: the seed medium consisted of 30 g·L^−1^ potato starch, 4.5 g·L^−1^ yeast extract, and 10.5 g·L^−1^ peptone.

Solid-state fermentation (SSF) medium was prepared based on Zeng et al. (2011), Liu et al. (2013), and Liu and Liao (2015) with certain modifications: the SSF medium consisted of 3 g dry wood chips and 4.5 ml nutrient solution in 250 ml conical flask, and the nutrient solution contained the following: glucose, 30 g·L^−1^; ammonium tartrate, 15 g·L^−1^; KH_2_PO_4_, 1 g·L^−1^; Na_2_HPO_4_, 0.2 g·L^−1^; MgSO_4_, 0.5 g·L^−1^; MnSO_4_, 0.034 g·L^−1^.

### Isolation and identification of pigment-producing fungi

Fragments of the basidiomata were treated as described in Zeng et al. (2011) and cultured on PDA plate at 30°C for 12 days. The strains with good growth and noticeable color change were selected for shaker screening.

For shaker screening, 10 ml of sterile normal saline (0.9% w·v ^−1^) were added to PDA plates covered with mycelium. The spores were scraped off and suspended in sterile normal saline to approximately10^8^ spores·mL^−1^. Each 1 ml of spore suspension was inoculated into 250 conical flasks containing 50 ml seed medium and cultured at 30°C, 200 r·min ^−1^ for 1 days in the dark. Then, 1 ml of seed medium was transferred into the SSF medium at 30°C for 12 days in the dark. The strain with high pigment yield was selected for subsequent studies.

The morphological characteristics were evaluated using a scanning electron microscopy (Quanta-200, FET, Netherlands) after 5 days of incubation on PDA plates (Wang et al., 2021). The molecular identification process was performed as follows: (1) The isolated strain was cultured for 5 days and harvested by sterilized spear tips; (2) after drying and grinding of the collected mycelium using liquid nitrogen, total genomic DNA was extracted using the CTAB protocol; (3) PCR amplification was performed as described by Saravanan et al. (2020), and the primers used were ITS1 and ITS4; (4) The ITS-5.8S rRNA gene sequence of the isolated strain was compared with the sequences deposited in the GenBank database, and phylogenetic tree was constructed by neighbor-joining method using MEGA 6.0 based on a bootstrap test of 1,000 replicates (Zeng et al., 2011; Huang et al., 2020).

### Substrate and solid-state fermentation

Here, five common agro-industrial wastes, such as sugarcane bagasse, wheat straw, rice straw, water hyacinth, and wood chip, were chosen to evaluate the growth and pigment production of isolated strain. Each of these five common agro-industrial wastes was used instead of the substrate (dry wood chips) in the original SSF medium, respectively, and the other components of the original SSF medium were unchanged. Additionally, inoculum preparation, inoculum size, and culture conditions were consistent with shaker screening of section “Isolation and Identification of Pigment-Producing Fungi.”

### Optimization of pigment production

According to the method described in Darwesh et al. (2020) and Chen et al. (2021), the “one factor at a time” design was used to assess the effect of different cultural conditions. These variables were different extra carbon sources (Monosaccharide: glucose and fructose; Disaccharide: sucrose and maltose; Polysaccharide: starch and β-cyclodextrin), nitrogen sources (Organic nitrogen: peptone, yeast extract, and carbamide; Inorganic nitrogen: ammonium nitrate, potassium nitrate, ammonium chloride, and ammonium tartrate), initial pH values (3, 4, 5, 6, 7, 8, 9, and 10), and relative humidity values (40, 45, 50, 55, 60, 65, 70, 75, and 80%). All the optimization experiments were performed in 250 ml conical flasks, and the SSF medium was prepared based on the section “Substrate and solid-state fermentation.” Unless otherwise indicated, the inoculum preparation, inoculum size, and culture conditions were consistent with shaker screening of section “Isolation and Identification of Pigment-Producing Fungi.”

### Biomass estimation

The biomass was estimated by measuring the N-acetylglucosamine released by the acid hydrolysis of the chitin, present in the cell walls of fungi. Sample handling and detection processes were according to the method described by Velmurugan et al. (2011). In brief, 0.5 g of dry fermented solid-state powder was first mixed with 1 ml of concentrated H_2_SO_4_. Then, 1 ml of acetylacetone reagent was added to the mixture and placed in a water bath at 100°C for 20 min. After cooling, 6 ml of ethanol and 1 ml of Ehrlich reagent were added successively and incubated at 65°C for 10 min. After cooling to room temperature, optical density (OD) was measured at 530 nm against the reagent blank using N-acetylglucosamine as the external standard.

### Pigment identification and estimation

Pigment extraction: After 10 d of incubation, the fermented solid substrate was dried to constant weight at 60°C in a cabinet and ground to a fine powder using a muller. 0.3 g of dry fermented solid-state powder was mixed with 15 ml of methanol and placed in a water bath at 35°C for 1 h, and this extraction was repeated twice (Luo et al., 2013). Then the extracts were filtered through Whatman filter paper Grade No. 1 at constant volume of 30 ml.

UV–visible spectroscopy: The production of pigments was estimated by detecting λmax of pigment extract (Chen et al., 2021). The maximum absorption peak of pigment extract was performed on the UV–visible U-3000 Spectrophotometer (Hitachi, Japan) with a scanning wavelength near 350 nm to 550 nm (Cruz-Munoz et al., 2015).

Chromatography: The method was according to that described by Dias and Urban (2009) and Kakoti et al. (2022) with certain modifications. In brief, chromatographic separation was performed on a BEH AMIDE column (1.7 μm, 2.1 mm × 100 mm; Waters, United States) using the ACQUITY Ultra Performance Liquid Chromatography system (Waters, United States). The column was maintained at 45°C and eluted with the gradient % A (solvent A): T (time, min) 5:0; 60:7; 100:9; 100:12; 5:15 at a flow rate of 0.3 ml·min^−1^. Solvent A was 100% acetonitrile, and B was 0.1% formic acid. The detection wavelength was 254 nm, and 5 μl of testing samples (methanol extraction and cinnabarinic acid standard) were injected into the column. All testing samples were filtered with nylon membrane (0.22 μm) before chromatographic separation.

Mass spectrometry: The mass spectrometry method was referred to that described by Xu et al. (2020) with certain modifications: mass spectrometry was performed on a Waters Maldi Synapt Q-TOF MS (Waters, US) operating in ESI^+^ ion modes. The desolvation gas was set to 300 l·h^−1^ at 300°C. The cone gas was set to 500 l·h^−1^ and the source temperature was set to 100°C. The capillary voltage and cone voltage were set to 3.5 kV and 30 V, respectively. The collision energy was set to 6 eV. The detector voltage was set to 1.7 kV and the scan range was from 50 to 2000 m·z^−1^.

Pigment estimation: Following the method of Velmurugan et al. (2011), the extracted pigment was quantified by measuring optical density (OD) at λmax and pigment yield was calculated by OD/gdfs. The gdfs represents per gram of dry fermented substrate. Methanol extract of the unfermented sample was used as the blank for pigment analysis.

### Assessment of antimicrobial activity

Agar plate diffusion assay was used to determine the antibacterial activity of the pigment produced by strain SYBC-L7 using *Bacillus subtilis* SYBC-hb1, *Bacillus subtilis* SYBC-hb5, *Bacillus licheniformis* SYBC-hb2, *Bacillus pumilus* SYBC-hb4, *Bacillus thuringiensis* SYBC-hb7, *Bacillus amyloliquefaciens* Y1-A3, *Bacillus megaterium* H021-A1, *Bacillus cereus* SYBC-hb8, *Lysinibacillus* sp. H021-S8, *Staphylococcus kloosii* H008-B4, and *Staphylococcus aureus* (Gram-positive bacteria) and *Escherichia coli* J159, *Serratia* sp. L1, *Serratia* sp. L2, and *Serratia* sp. L3 (Gram-negative bacteria). The indicator strains were incubated at 37°C in Luria-Bertani (LB) broth. After 18 h of incubation, the cultures were diluted to 10^7^ cfu·mL^−1^ and “flood-inoculated” onto the surface of LB plates. 9 mm diameter wells were cut using a punching bear. Then, 100 μl of methanol extract of pigment with different concentrations (pigment extraction:5, 10, 15, 20 OD·gdfs^−1^, containing CA concentrations of 21.6, 43.2, 64., and 86.3 mg·L^−1^, respectively) were delivered into the wells with pure methanol as blank and different concentrations (50, 100, 150, 200, 300, 400, 500 mg·L^−1^) of ampicillin as reference. After incubation at 37°C for 18 h, plates were examined for any zones of growth inhibition and measured the diameter of each zone (subtracted the diameter of well).

### Statistical analysis

All the experiments were performed in triplicate, and the error bar represents the standard deviations (SD). OriginPro 2022b software was used to calculate the mean value/SD and to plot figures of the collected data.

## Results and discussion

### Isolation and Identification of Pigment-Producing Fungi

**Figure 1:**
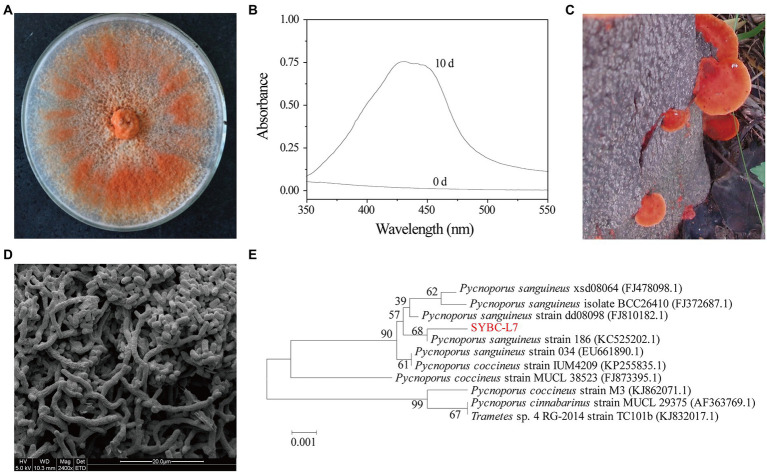
Orange pigment-producing fungal isolate (strain SYBC-L7) with the highest potency. **(A)** The mycelia of strain SYBC-L7 cultured on PDA medium for 9 d; **(B)** Spectral analysis of extract samples obtained from strain SYBC-L7 with wood chip for 10 d, and 0 d represent the unfermented solid substrate sample; **(C)** The fruit body of strain SYBC-L7; **(D)** Microscopic characteristics of the mycelia of strain SYBC-L7 cultured on PDA medium for 5 d; **(E)** Phylogenetic trees based on ITS-5.8S rRNA sequences of strain SYBC-L7 and others downloaded from NCBI.

Ten fungal fruit body samples collected from different locations were subjected to plate screening and shaker screening successively to select the most efficient strain in terms of pigment production. A fungal strain designated SYBC-L7 was clearly observed to rapidly produce an intense orange pigment with an absorption maximum at a wavelength of 430 nm (Figures 1A,B). Thus, strain SYBC-L7 was selected for the subsequent studies.

Strain SYBC-L7 was identified based on its morphological and molecular properties (Figure 1). The basidiocarp of strain SYBC-L7 was smooth, acute margin, smooth to wavy thin, pileus shortly, and sessile or sometimes overlapping with characteristic color of bright orange-red (Figure 1C). After inoculation to PDA plate and culture at 30°C, the mycelium of strain SYBC-L7 grew faster and gradually broke off to form a large number of spores, which are rectangular in shape, smooth in surface, and varied in size (Figure 1D); By the 9th day of culture, the mycelium covered full of the plate and was orange-red (Figure 1A). These characteristics were consistent with *Pycnoporus* genus (Tellez-Tellez et al., 2016). Moreover, the amplification of the genomic DNA of strain SYBC-L7 by primers ITS1 and ITS4 produced a single amplification product of approximately 639 bp. When comparing the sequence to the GenBank database and constructing the phylogenetic tree, it was observed that the sequence (GenBank ID: HQ891291.1) was clustering to *Pycnoporus sanguineus* genus (Figure 1E). As the sequence similarity to the most closely reference strain (GenBank ID: KC525202.1) was only 68%, strain SYBC-L7 may be a new strain of *Pycnoporus sanguineus*.

### Solid substrate chosen for solid-state fermentation

**Figure 2:**
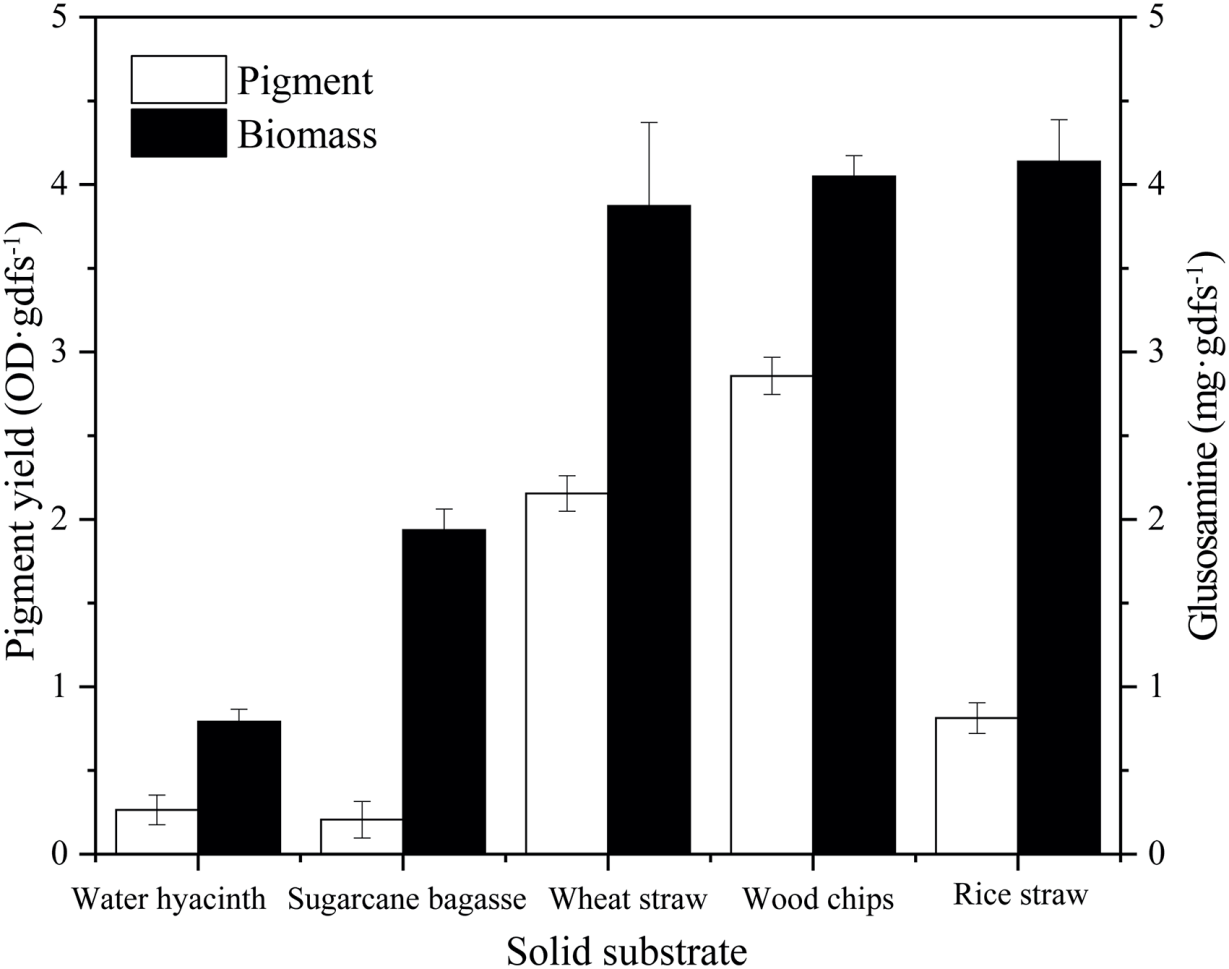
Effect of solid substrate on biomass and pigment production. Data correspond to the mean ± SD of three replicates.

In the solid-state fermentation process, the fermentation substrate has a great influence not only on the growth, attachment, and extension of the mycelium but also on factors such as heat dissipation and oxygen supply (Mussatto et al., 2012). Here, five common agro-industrial wastes (sugarcane bagasse, wheat straw, rice straw, water hyacinth, and wood chip) were selected to evaluate the growth and pigment production of strain SYBC-L7. As shown in Figure 2, the growth and pigment production of strain SYBC-L7 were greatly influenced by the used agro-industrial waste. Among the tested wastes, the wood chip was found to be the best substrate not only giving a maximum pigment yield but also greatly promoting the growth of strain SYBC-L7. In the literature, most *Pycnoporus* species have been isolated and used as enzyme producers (Tellez-Tellez et al., 2016), but few of them were used to produce pigment, which was shown in (Supplementary Table S1; Eggert, 1997; Smania et al., 1997; Cruz-Munoz et al., 2015; Sutthisa and Sanoamuang, 2017; Zhang et al., 2019). However, our study is the first report, to our knowledge, on using agro-industrial waste, especially wood chips as a culture substrate for pigment production by *Pycnoporus sanguineus* SYBC-L7.

### Optimization of pigment production by strain SYBC-L7

**Figure 3:**
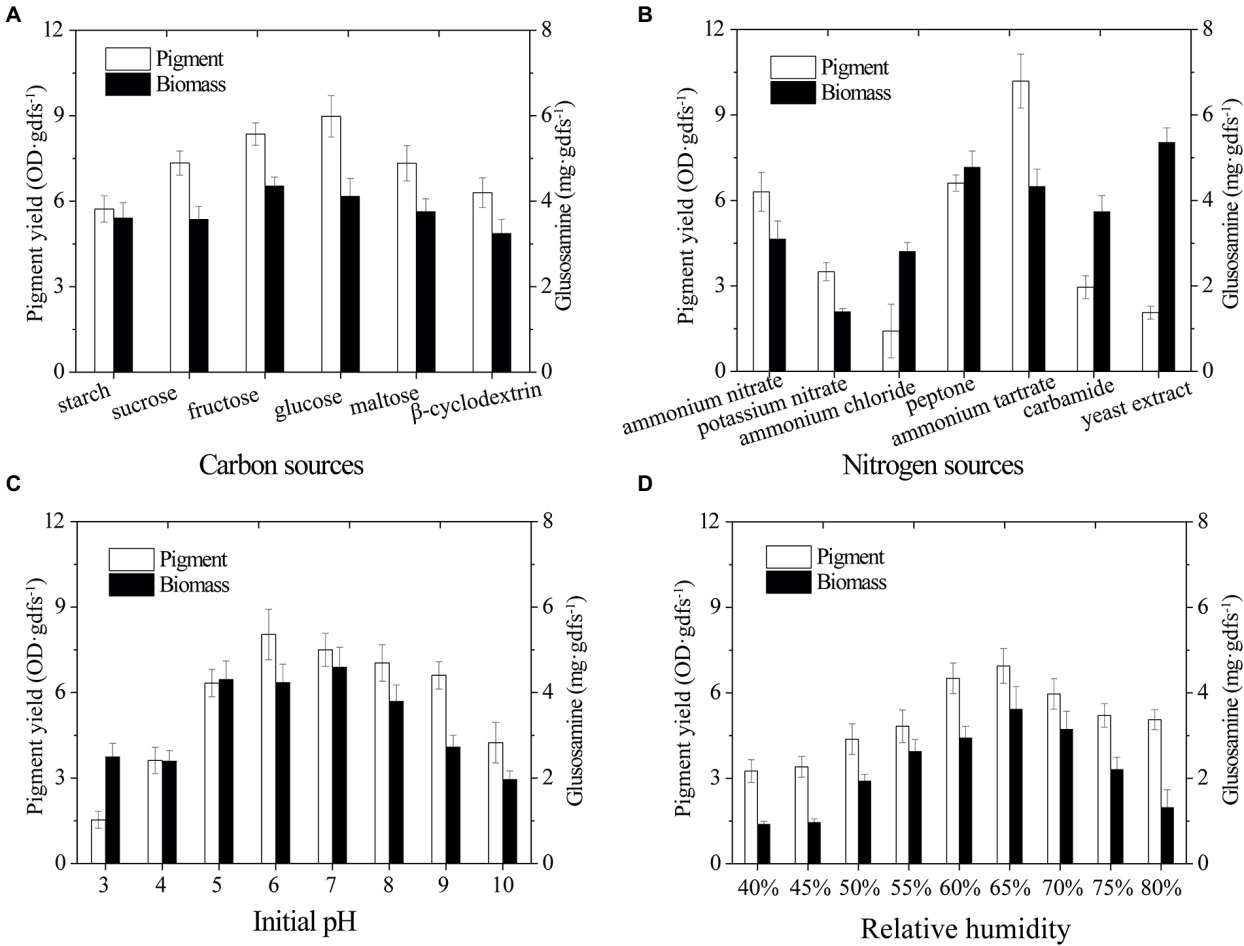
Effect of carbon sources **(A)**, nitrogen sources **(B)**, initial pH **(C)**, and relative humidity **(D)** on biomass and pigment production strain SYBC-L7 in solid-state fermentation. Data correspond to the mean ± SD of three replicates.

Biomass growth and pigment productivity can be influenced by nutrient fermentation conditions (Darwesh et al., 2020). Here, environmental and cultural conditions were studied to improve the yield of pigment produced by strain SYBC-L7. Carbon is not only an essential nutrient for the biosynthesis of cellular components but also an energy resource for cells (Feng et al., 2018). For example, carbon plays a critical role in cell growth, metabolism, and pigment production of *Monascus* spp. (Darwesh et al., 2020; Long et al., 2020). In the present study, different extra carbon sources (Monosaccharide: glucose and fructose; Disaccharide: sucrose and maltose; Polysaccharide: starch and β-cyclodextrin) were used to improve the biomass and pigment production of strain SYBC-L7. The results presented in Figure 3A showed that under the experimental conditions, monosaccharide was easier to be utilized for growth and pigment production than disaccharide and polysaccharide. Glucose was better for pigment synthesis, fructose was better for fungal growth, while, lower pigment production and biomass were obtained with β-cyclodextrin and starch. In accordance with our results, Zhao et al. (2019) and Shahin et al. (2022) stated that glucose was the optimum carbon source for pigment production.

Nitrogen source, another essential building component, can influence microbial growth and the production of bioactive metabolites (Tudzynski, 2014; Landi et al., 2019; Darwesh et al., 2020). To improve the pigment yield, different extra nitrogen sources (Organic nitrogen: peptone, yeast extract, and carbamide; inorganic nitrogen: ammonium nitrate, potassium nitrate, ammonium chloride, and ammonium tartrate) were added to the solid-state fermentation culture. As shown in Figure 3B, strain SYBC-L7 grew better and produced the most pigments with ammonium tartrate, and it grew better and produced more pigments with peptone. While it grew best but produced fewer pigments with yeast extract as an extra nitrogen source.

The initial pH of the culture medium is one of the most critical environmental and culture parameters determining microbial growth and metabolic activity (Ouyang et al., 2020). As different strains have different optimum pH, here, an experiment was conducted to determine the effect of different initial pH values (3–10) on the biomass and pigment yield of strain SYBC-L7. As shown in Figure 3C, high pigment production was obtained at pH 5 to 9, and the maximum production was received at pH 6. Low or high pH (like 3, 4, and 10) inhibited both biomass and pigment production. The results were similar to those of Cruz-Munoz et al. (2015), who reported that the maxima pigment synthesis was obtained at pH 7 for *Pycnoporus sanguineus* strain H1 and H2.

The relative humidity of the incubator is also one of the most critical environmental parameters of the SSF process. It can prevent accelerated drying of the fermentation substrate, and it is directly related to water activity, which is a critical factor for fungal metabolism performance during the fermentation process (Osorio et al., 2020). As shown in Figure 3D, maximum pigment yield and biomass were observed at 65% of relative humidity, and a decrease in pigment yield was observed below or above 65%. The humidity gradient occurred between the exterior surface and inner surface of substrate (He et al., 2019). Low relative humidity could accelerate the formation of this humidity gradient, promote liquid water movement and evaporation from the interior to the surface of substrate, and lead to low nutrient availability and less efficient heat exchange, causing poor pigment yield (Babitha et al., 2007; He et al., 2019). On the contrary, the higher relative humidity could promote moisture in the air movement from the surface to the interior of substrate, and reduce the mass transfer process, air transfer, and extension of mycelium in SSF, leading to suboptimal pigment formation (Babitha et al., 2007).

### Identification of pigment from strain SYBC-L7

**Figure 4:**
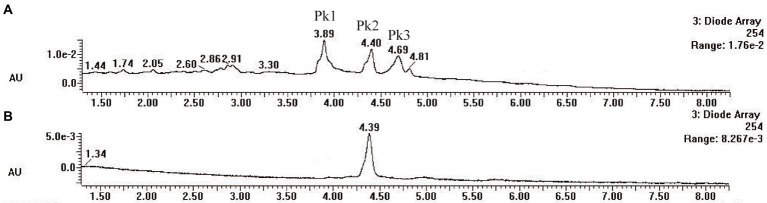
HPLC analysis of pigment extracts of strain SYBC-L7. **(A)** HPLC chromatograms of pigment extracts of strain SYBC-L7; **(B)** HPLC chromatograms of CA standard sample.

*Pycnoporus sanguineus* is one of the promising organisms for developing natural pigments, and it could produce various shades of red, orange, yellow, and brown color (Zhang et al., 2019). These primary or secondary metabolites produced by *Pycnoporus* genus are different and depend on the species and culture conditions (Cruz-Munoz et al., 2015; Tellez-Tellez et al., 2016). Nevertheless, previous studies have demonstrated that cinnabarin and cinnabarinic acid (CA) are the main pigment components of *Pycnoporus* strains, which have antiviral, antibacterial, and anti-inflammatory activity (Tellez-Tellez et al., 2016). In this study, CA was taken as a standard sample and used for pigment component analysis. In Figure 4, three obvious strong peaks (marked as Pk1, Pk2, and Pk3) appeared at the retention time (RT) of 3.89 min, 4.40 min, and 4.69 min, respectively. The mass spectrum of Pk2 showed a molecular ion at m/z 301 [M + H]^+^ (Supplementary Figure S1), which was not only the same as described in some previous studies (Dias and Urban, 2009; Tellez-Tellez et al., 2016), but also was consistent with the mass spectrum of CA standard sample (data was not shown). Thus, Pk2 was identified as *CA.* The protonated ions of Pk1 and Pk3 were both at m/z 285 [M + H]^+^ (Supplementary Figures S1), and wavelength scanning results showed that their characteristic absorption peaks were both between 430 and 450 nm (Supplementary Figure S2); These results are in fair agreement with the results reported for tramesanguin (Dias and Urban, 2009). As Pk1 and Pk3 are different compounds, Pk1 and Pk3 maybe tramesanguin and its isomer, 2-amino-9-formylphenoxazone-1-carbonic acid. The structures of these compounds are shown in Table 1.

**Table 1:** Identification of pigment from strain SYBC-L7.

Products	RT (min)	Measured mass [M + H]^+^ (m/z)	Common name	Proposed structure	References
Pk2	4.40	301	Cinnabarinic acid	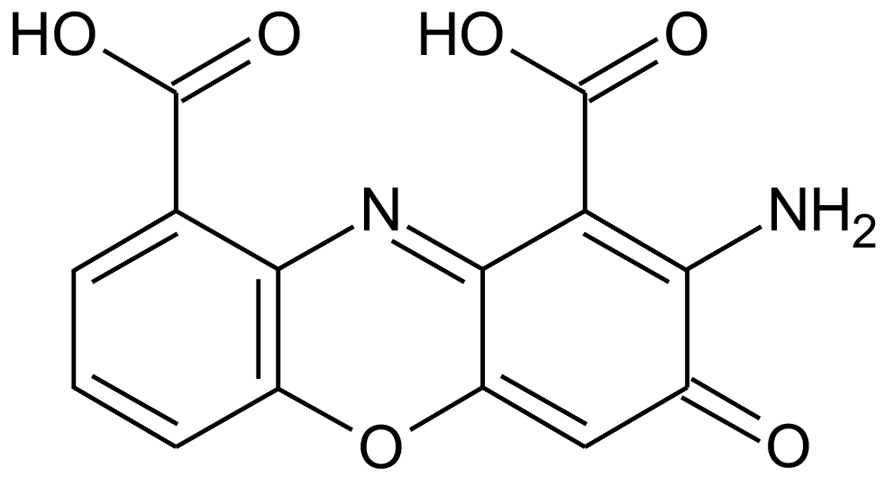	Dias and Urban (2009), Tellez-Tellez et al. (2016)
Pk1	3.89	285	Tramesanguin, 2-amino-9-formylphenoxazone-1-carbonic acid	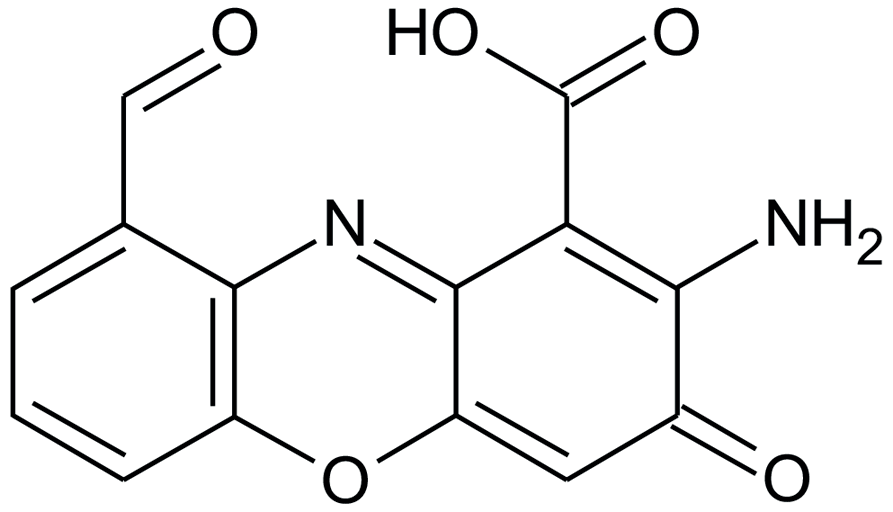	Dias and Urban (2009)
Pk3	4.69	285			

### Antibacterial activity analysis of pigment extract

**Figure 5:**
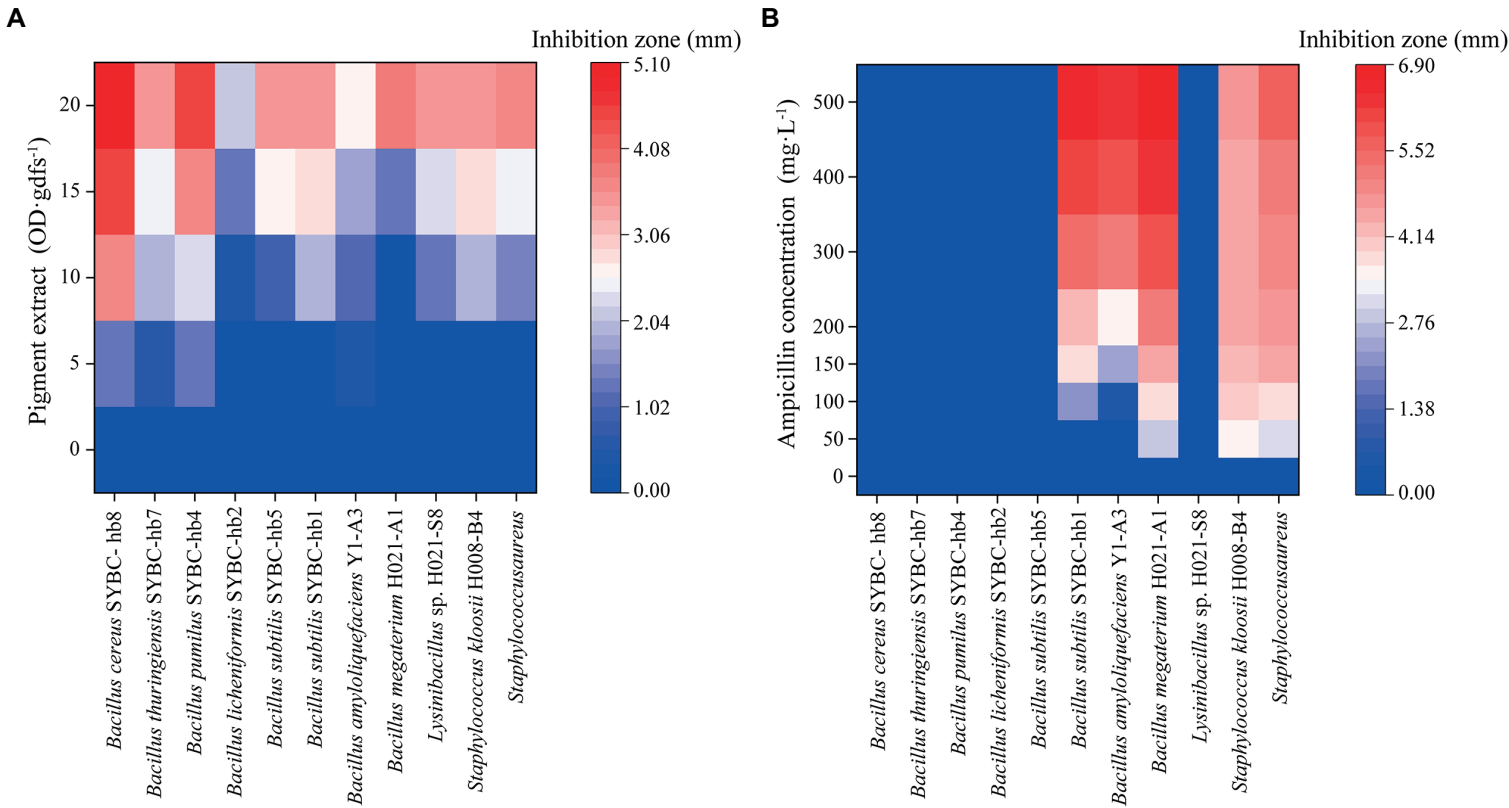
Heatmaps of antibacterial activity of pigment extract **(A)** and ampicillin **(B)** for Gram-positive bacteria. Data correspond to the mean of three replicates.

The antibacterial activity of pigment extract was evaluated by agar plate diffusion assay. In agar plate diffusion assay, pigment extract exhibited significant antibacterial activity on Gram-positive bacteria (Figure 5A), and the zone of inhibition was increased with the increase of pigment concentration. However, pigment extract exerted no inhibitory effect on Gram-negative bacteria (data not shown). These findings were similar to those of Tellez-Tellez et al. (2016), who reported that the component from *Pycnoporus sanguineus* showed more activity against Gram-positive than Gram-negative bacteria. Kakoti et al. (2022) reported that CA extracted from *Trametes coccinea* fruiting bodies showed high inhibition for Gram-negative bacteria, and the minimal inhibitory concentration of CA for *Escherichia coli* was 300 mg·L^−1^. The pigment extract (containing CA concentrations of 21.6, 43.2, 64.7, and 86.3 mg·L^−1^, respectively) in our study showed no inhibitory effect on Gram-negative bacteria including *Escherichia coli*, which may be because of the low concentration of pigment extract. Additionally, when compared with the antibacterial activity of different concentrations of ampicillin (50–400 mg·L^−1^) on Gram-positive bacteria, the pigment extract had an inhibitory effect on all 11 kinds of indicator bacteria, while ampicillin only had an inhibitory effect on five of them (like *Bacillus subtilis* SYBC-hb1, *Bacillus amyloliquefaciens* Y1-A3, *Bacillus megaterium* H021-A1, *Staphylococcus kloosii* H008-B4, *Staphylococcus aureus*; Figure 5B).

## Conclusion

A new fungal strain *Pycnoporus sanguineus* SYBC-L7 was shown to effectively produce intense orange pigments in solid-state fermentation. Pigment production processes could be more economic and high yield by controlling agro-industrial wastes as substrates and fermentation conditions such as carbon sources, nitrogen sources, initial pH, and relative humidity. The pigment extract was identified as cinnabarinic acid, tramesanguin, and 2-amino-9-formylphenoxazone-1-carbonic acid by HPLC-MS. Additionally, antibacterial activity analysis of pigment extract produced by strain SYBC-L7 showed significant antibacterial activity on different bacteria, and the pigment was more sensitive to Gram-positive bacteria, indicating its potential application for areas such as the food, cosmetics, nutraceuticals, and textile industry that need color. Further studies should be conducted to better understand the biosafety of microbial pigments as promising alternatives to hazardous artificial colorants.

## Data availability statement

The original contributions presented in the study are included in the article/Supplementary material, further inquiries can be directed to the corresponding authors. The ITS-5.8S rRNA gene sequence of Pycnoporus sanguineus SYBC-L7 is available in the NCBI database, accession number HQ891291.1.

## Author contributions

DM and XS participated in data analysis and wrote the paper. S-PL, Q-PT, and X-RL performed the research. All authors contributed to the article and approved the submitted version.

## Funding

This work was financially supported by startup funds from Shangqiu Normal University (grant no. 7001/700219), horizontal subject (grant no. 8001/801141), and the project fund from the Program for Science and Technology Innovative Research Team in University of Henan Province (grant no. 21IRTSTHN025).

## Conflict of interest

S-PL was employed by Hua Tian Engineering & Technology Corporation, MCC, Nanjing, China.

The remaining authors declare that the research was conducted in the absence of any commercial or financial relationships that could be construed as a potential conflict of interest.

## Publisher’s note

All claims expressed in this article are solely those of the authors and do not necessarily represent those of their affiliated organizations, or those of the publisher, the editors and the reviewers. Any product that may be evaluated in this article, or claim that may be made by its manufacturer, is not guaranteed or endorsed by the publisher.
