# Supplementary material for: Pigment production by a newly isolated strain Pycnoporus sanguineus SYBC-L7 in solid-state fermentation
Source: Front Microbiol. 2022 Oct 19;13:1015913. doi: 10.3389/fmicb.2022.1015913 (PMC9628674; doi:10.3389/fmicb.2022.1015913)
Supplement: Supplementary file 1 [file Data_Sheet_1.docx]

**Supplementary materials**


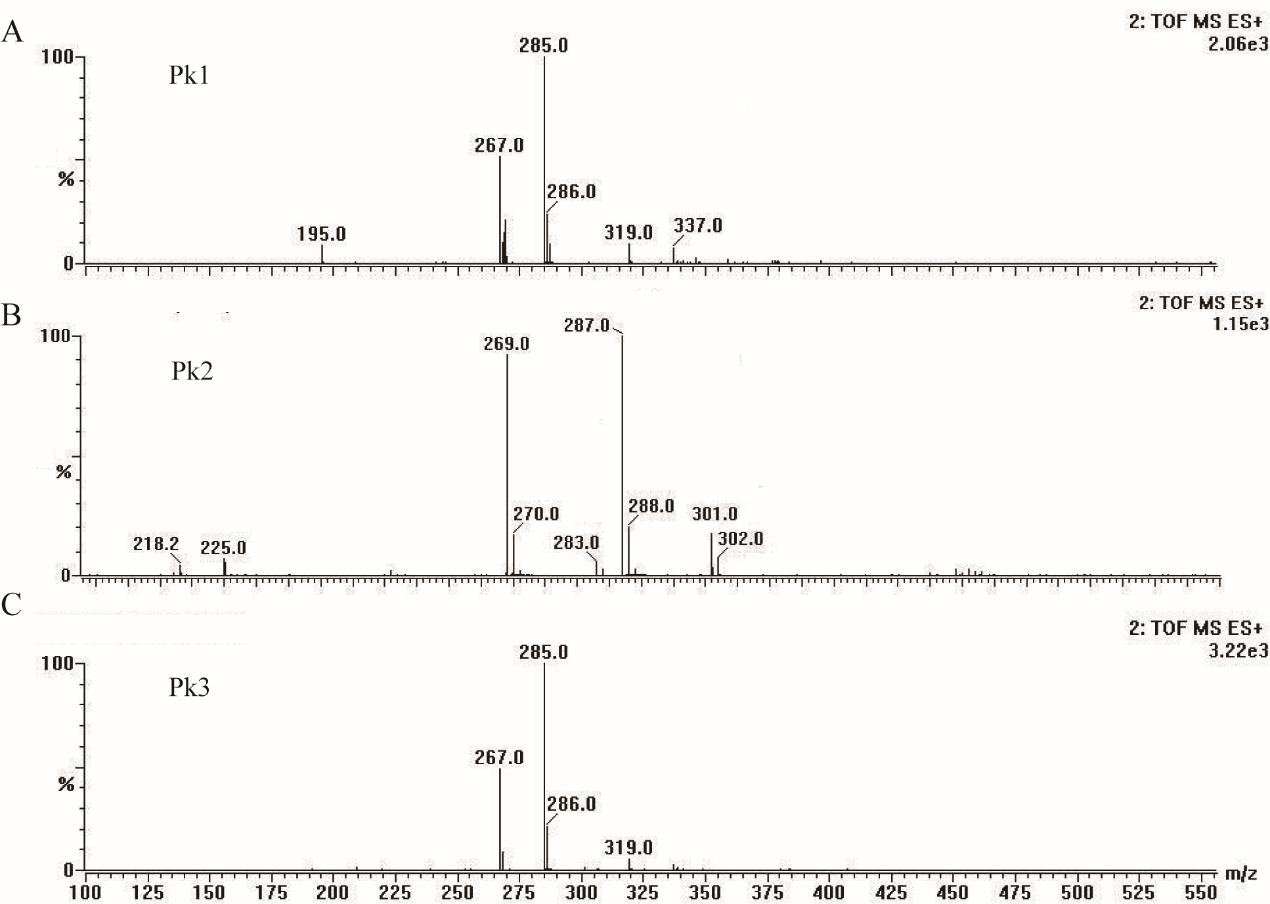


FIGURE S1 Mass spectra of pigment extraction. A, mass spectra of Pk1 at 3.89 min; B, mass spectra of Pk2 at 4.40 min; C, mass spectra of Pk3 at 4.69 min.


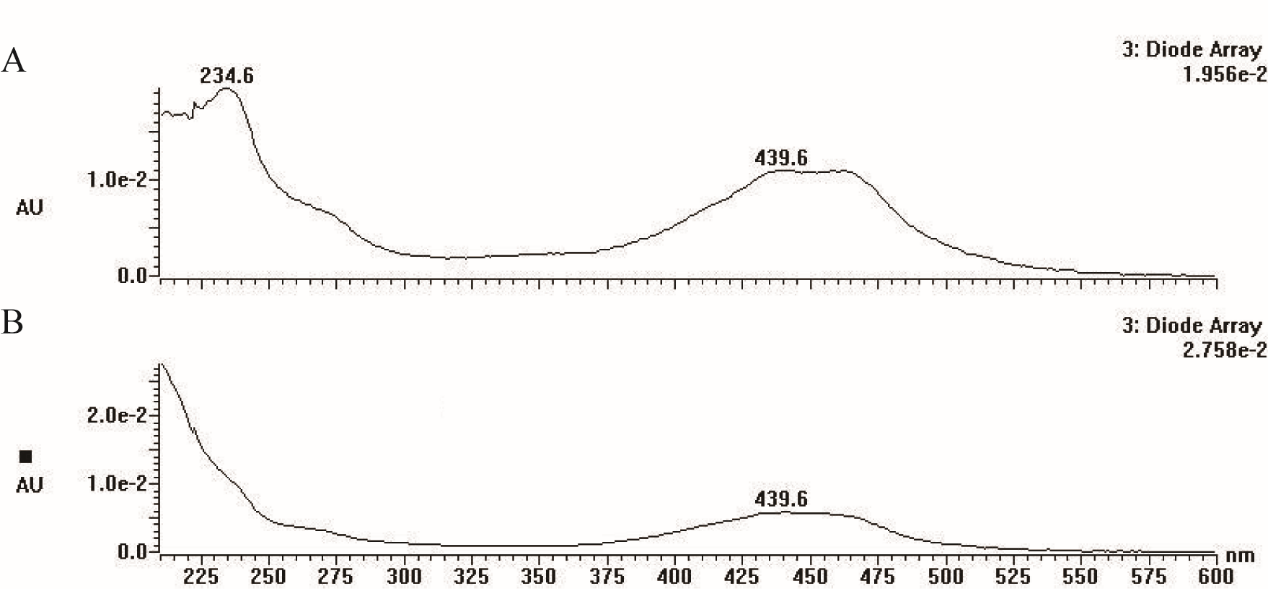


FIGURE S2 Wavelength scanning of pigment by UV-visible spectroscopy. A, pigment extraction at Pk1; B, pigment extraction at Pk3.

TABLE S1 Summary of the pigment-production potential of different *Pycnoporus* species

| Microorganism | Source | Culture conditions | Pigment yield | References |
| --- | --- | --- | --- | --- |
| *Pycnoporus sanguineus* strain 28cc | Fenglin National Nature Reserves of Heilongjiang  province, China | PDA liquid medium, at 28 °C for 15d | No report | Zhang et al., 2019 |
| *Pycnoporus coccineus* KKUPN1 | Khon Kaen University, Thailand | Potato dextrose broth containing 0.05 and 0.1% (v/v) colchicine, at 28 °C for 7 d | No report | Sutthisa et al., 2017 |
| *Pycnoporus sanguineus* H1 | Casuarina trees, Mexico | PDA solid medium, pH 7.0, 23±3 °C, under light for 30 d | 17.5 mg·L^-1^ | Cruz-Munoz et al., 2015 |
| *Pycnoporus sanguineus* H2 | Mango trees, Michoacan | PDA solid medium, pH 7.0, 23±3 °C, under light for 30 d | 10.8 mg·L^-1^ | Cruz-Munoz et al., 2015 |
| *Pycnoporus cinnabarinus*  ATCC 200478 | No report | Basal medium with glucose as carbon source, at 24 °C for 9 d | No report | Eggert et al., 1997 |
| *Pycnoporus sanguineus* MIP 89007 | Collected in the state of Santa Catarina,  Southern Brazil | Potato dextrose broth, pH 9.0, 25 °C, under light for 20 d | No report | Smania et al., 1997 |
